# Cost-Effectiveness of Including a Nurse Specialist in the Treatment of Urinary Incontinence in Primary Care in the Netherlands

**DOI:** 10.1371/journal.pone.0138225

**Published:** 2015-10-01

**Authors:** K. M. Holtzer-Goor, J. G. Gaultney, P. van Houten, A. S. Wagg, S. A. Huygens, M. M. J. Nielen, C. P. Albers-Heitner, W. K. Redekop, M. P. Rutten-van Mölken, M. J. Al

**Affiliations:** 1 Erasmus University Rotterdam, Institute for Medical Technology Assessment / Institute of Health Policy and Management, Rotterdam, The Netherlands; 2 Zonnehuisgroep Amstelland, Medical department, Amstelveen, the Netherlands; 3 University of Alberta, Department of Medicine, Edmonton, AB, Canada; 4 NIVEL (Netherlands Institute for Health Services Research), Department of General Practice, Utrecht, the Netherlands; 5 Maastricht University Medical Center, Department of Obstetrics and Gynecology, Maastricht, the Netherlands; TNO, NETHERLANDS

## Abstract

**Objective:**

Incontinence is an important health problem. Effectively treating incontinence could lead to important health gains in patients and caregivers. Management of incontinence is currently suboptimal, especially in elderly patients. To optimise the provision of incontinence care a global optimum continence service specification (OCSS) was developed. The current study evaluates the costs and effects of implementing this OCSS for community-dwelling patients older than 65 years with four or more chronic diseases in the Netherlands.

**Method:**

A decision analytic model was developed comparing the current care pathway for urinary incontinence in the Netherlands with the pathway as described in the OCSS. The new care strategy was operationalised as the appointment of a continence nurse specialist (NS) located with the general practitioner (GP). This was assumed to increase case detection and to include initial assessment and treatment by the NS. The analysis used a societal perspective, including medical costs, containment products (out-of-pocket and paid by insurer), home care, informal care, and implementation costs.

**Results:**

With the new care strategy a QALY gain of 0.005 per patient is achieved while saving €402 per patient over a 3 year period from a societal perspective. In interpreting these findings it is important to realise that many patients are undetected, even in the new care situation (36%), or receive care for containment only. In both of these groups no health gains were achieved.

**Conclusion:**

Implementing the OCSS in the Netherlands by locating a NS in the GP practice is likely to reduce incontinence, improve quality of life, and reduce costs. Furthermore, the study also highlighted that various areas of the continence care process lack data, which would be valuable to collect through the introduction of the NS in a study setting.

## Introduction

Incontinence, whether urinary or faecal, is a significant health problem worldwide that has a negative impact on the health and quality of life of patients and their caregivers. In most studies including adults from all over the world, prevalence rates vary between 11 and 15% for faecal incontinence and 25% and 45% for urinary incontinence [1]. In the Netherlands, an estimated 800,000 people have some level of incontinence [2], although the actual number could be higher due to reluctance to seek help. For many people incontinence is a taboo topic that they find difficult to discuss, even with their general practitioner (GP). People may also not talk about incontinence because it is thought to be inherent to ageing or because they are unaware of available treatments [3, 4]. Many people appear to have suffered from incontinence for a long time prior to the first visit to the GP [3].

Both urinary and faecal incontinence are most common in older persons. However, urinary incontinence (UI) is far more common with a ratio of 6:2:1 for UI versus faecal incontinence (FI) versus both [3]. Each year in the Netherlands, approximately 64,000 new patients report to the doctor with UI [5]. In older people, UI greatly influences quality of life since it is often accompanied by feelings of shame, depression and low self-esteem. It is also a risk for falls and is associated with admission to a nursing home [6, 7]. Unfortunately, studies show that, especially in older patients, care for UI is below standard [8–11]. It is therefore important that further efforts be made to ensure that elderly people receive the best care available.

Besides the practical, hygienic and social problems experienced by people with UI, its chronic nature has a negative impact on the psychological health of caregivers [12, 13] and is also associated with high costs for health care and society [14]. The economic costs of incontinence absorbing material, diagnostic tests, physiotherapy, surgical procedures and work loss have been shown to be substantial [15–18]. In 2000, the direct and indirect costs of urinary incontinence were $19.0 billion and $0.5 billion in the USA, respectively [16]. The direct annual medical costs of urinary incontinence per inhabitant (€71) are similar to those of coronary heart disease (€78), and higher than the costs of diabetes or refraction errors/accommodation problems [19].

To improve the standard of care delivery for UI and FI in community dwelling patients and their health, an optimum continence service specification was developed for use internationally, which aimed to make recommendations on service delivery that would enable care specified in most national and international clinical guidelines to be delivered [1, 20–22]. The optimum continence service specification adopted a modular approach that could be adapted to specific local circumstances [23]. The specification included case detection, initial assessment and treatment, case co-ordination, caregiver support, community-based support, specialist assessment and treatment, use of containment products and use of technology.

In the optimum continence service specification four profiles of patients were explored in detail: stress and urgency UI, faecal incontinence, incontinence because of neurological diseases, and elderly/cognitively impaired. Note that these groups are not necessarily mutually exclusive. Given the great heterogeneity between the four profiles, one was initially selected for the purposes of testing the service specification, i.e. elderly/cognitively impaired. This patient profile was chosen because this population was deemed to have a greater unmet need than patients in the other profiles. It was operationalised as elderly (>65 years) patients with four or more co-existent chronic diseases. We focused on UI, because there was insufficient information available to conduct the analysis for FI. The patient group we selected often experiences restrictions in their mobility or cognitive functioning, thereby increasing the number of incontinence episodes. Evidence suggests that these individuals may receive a lower quality of care than younger people [24, 25]. Moreover, this group of patients already visits the GP practice for other reasons than incontinence, making them ideally suited for case finding and deploying follow-up strategies. Additionally, this population is expected to increase further in future decades.

Alongside improved health and quality of life of incontinent patients, adoption of the optimum continence service specification could also have an impact on the costs of care. For example, active detection could lead to increased costs of treatment. At the same time, an increase in patients successfully treated for incontinence may reduce the costs of medication, containment products, formal home care use and informal care use. Also, adverse events related to incontinence such as falls, incontinence related skin damage and urinary tract infections may be prevented. Therefore, an economic evaluation was carried out to assess the cost-consequences and health effects of the possible implementation of the optimum continence service specification in the Netherlands compared with the current situation.

## Patients and Methods

### Optimum continence service specification

The implementation of the continence service specification for UI in the Netherlands included the following changes to the current delivery of care: 1) more active *case detection* in primary care by either the primary care physician or a continence nurse specialist (NS); 2) *initial assessment and treatment* by a continence NS, potentially leading to more successfully treated patients and more patient and carer centred prescription of containment products; and 3) improved *case coordination* by NSs who will liaise with home care agencies (agencies that provide personal care, [specialised] nursing care, instructions and information, and guidance and support to patients at home) and both formal and informal caregivers to address the patient’s medical and non-medical care needs [23]. The type of continence nurse specialist proposed in this evaluation is someone who is allowed to prescribe drugs and containment products, refer patients for specialist care and perform physical examinations of the patients.

### Patient population

The population of community-dwelling patients older than 65 years with four or more chronic diseases was defined using a published list of chronic diseases based on ICPC-2 codes (but recoded to ICPC-1) [26]. This list includes morbidities such as hypertension and obesity, which might be seen more as risk factors rather than diseases. Combinations of diseases that are common among elderly in the general population are: hypertension, osteoarthritis, ischaemic heart disease, obesity, atherosclerosis and diabetes [27]. Patients who were 65 years or older with fewer chronic diseases were not included in our analysis, since they are less dependent on formal and informal care.

The size of the target population was estimated by combining the percentage of eligible patients per age group and gender group [28] with the number of non-institutionalised Dutch elderly persons [29] per age and gender group (See Table 1). Incidence and prevalence rates for UI were those of 219 Dutch general practices participating in NIVEL Primary Care Database in 2012 [30] and the study of Teunissen et al. [3] respectively. UI cases in this group were defined as patients complaining of any involuntary loss of urine. Although UI varies in terms of severity and aetiology [1], all UI cases were included in the analysis.

**Table 1:** Characteristics of the target patient population.

Characteristics	Estimate	Source
Size of target patient population:		
Number of community-dwelling elderly in the Netherlands (65 or older)	2,591,357	CBS [29]
% patients with 4 or more chronic diseases corrected for age distribution	31%	NIVEL [30]
**Total number of community-dwelling elderly with ≥4 chronic diseases**	**808,503**	
Prevalence and incidence of UI in target patient population:		
Yearly incidence rate for UI in elderly with **≥4** chronic diseases	3.20%	NIVEL [30]
**Total number detected incident cases UI in elderly with ≥4 chronic diseases per year**	**25,872**
% of elderly patients with self-reported symptoms of UI	25%	Teunissen [3]
**Total number prevalent cases UI in elderly with ≥4 chronic diseases per year**	**58306**
**Men**	**13,410 (23%)**	CBS [29], Uijen [28], Teunissen [3]
**Women**	**44,896 (77%)**	CBS [29], Uijen [28], Teunissen [3]

### Analytical approach

**Fig 1:**
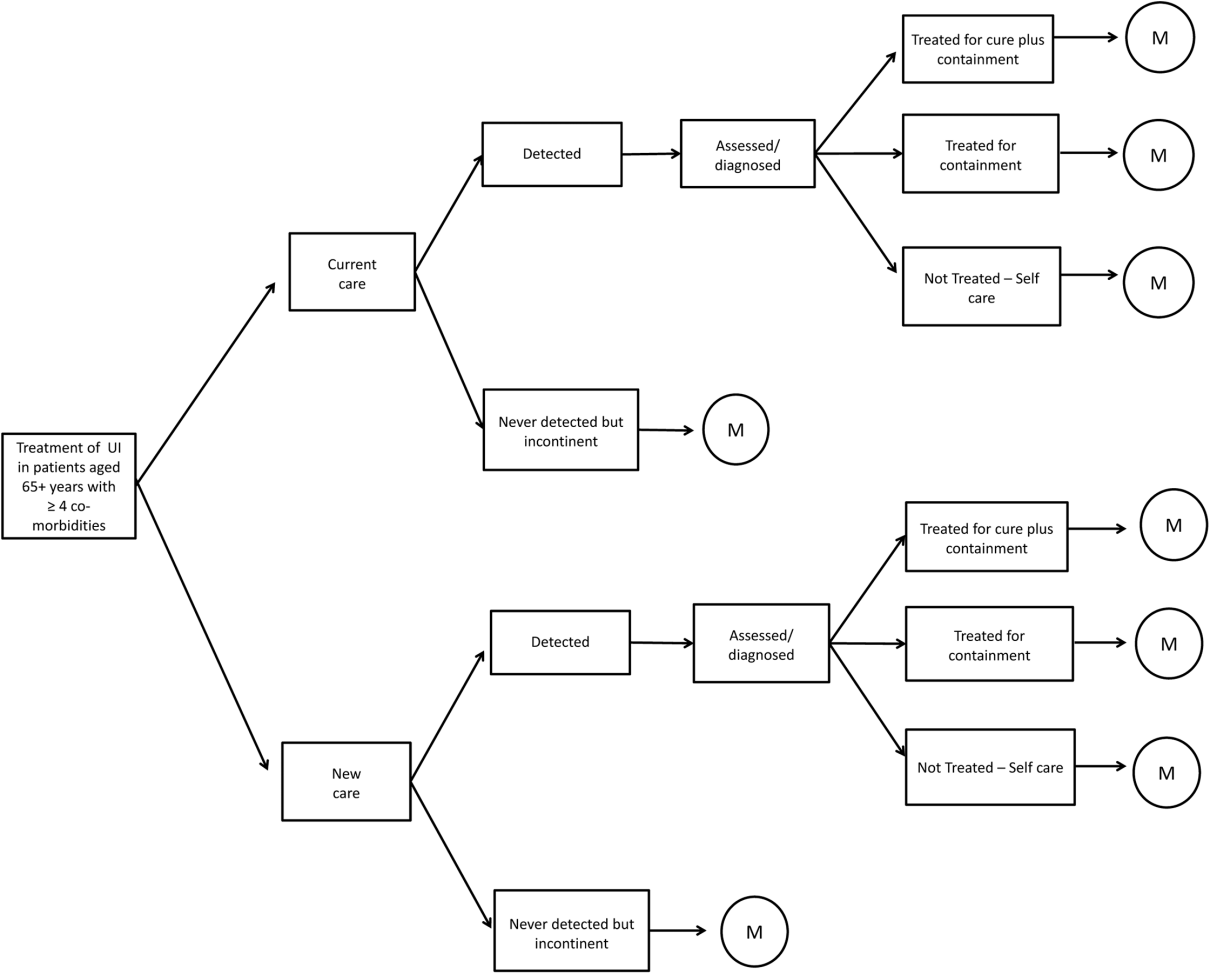
Decision analytic model structure. *M denotes Markov model structure.

To calculate the cost-effectiveness of the implementation of the optimum continence service specification, a decision analytical model was developed using Excel (Fig 1). The aim of the model was to compare the total costs and health benefits of the current standard of care (current care) for UI with an alternative standard of care approach that incorporates the optimum continence service specification (new care). The new approach to care consists of more active detection of patients with UI and the initial assessment and treatment by a nurse continence specialist (NS). The potential improvement in tailoring the prescribed containment products to the patient’s and carer’s needs and the potential improvement in case coordination were not included in the current evaluation, as no studies were retrieved that quantified such effects. The calculations of costs and health benefits were limited to a time horizon of 3 years. The model was also used to perform a 3-year budget impact analysis.

### Model structure

The structure of the decision analytic model was developed to reflect the entire pathway for UI care in the Netherlands. All possible care pathways were identified by means of a series of interviews conducted with healthcare experts: 3 GPs, 3 pelvic physiotherapists, 2 continence nurses, 3 gynaecologists, 2 surgeons, 2 urologists, a geriatric specialist, a gastroenterologist, and a pharmacist. The entire care pathway begins with the detection phase, where patients can be detected by a GP or never be detected and remain incontinent. Patients who are never detected remain in this category for the entire duration of the model. All detected patients continue on to the initial assessment phase where the type and severity of incontinence is assessed. Initial assessment in the Netherlands can be performed by either a GP or pelvic physiotherapist [31]. Patients then move to the treatment phase where they can enter one of three care pathways: 1) treatment for cure alongside treatment for containment, where the aim is to reduce the number of incontinence episodes as much as possible; 2) treatment for containment only, where the patient receives a prescription for pad use but is not actively followed for improvement of symptoms; or 3) self-management, where the patient is neither actively treated for cure nor treated for containment.

**Fig 2:**
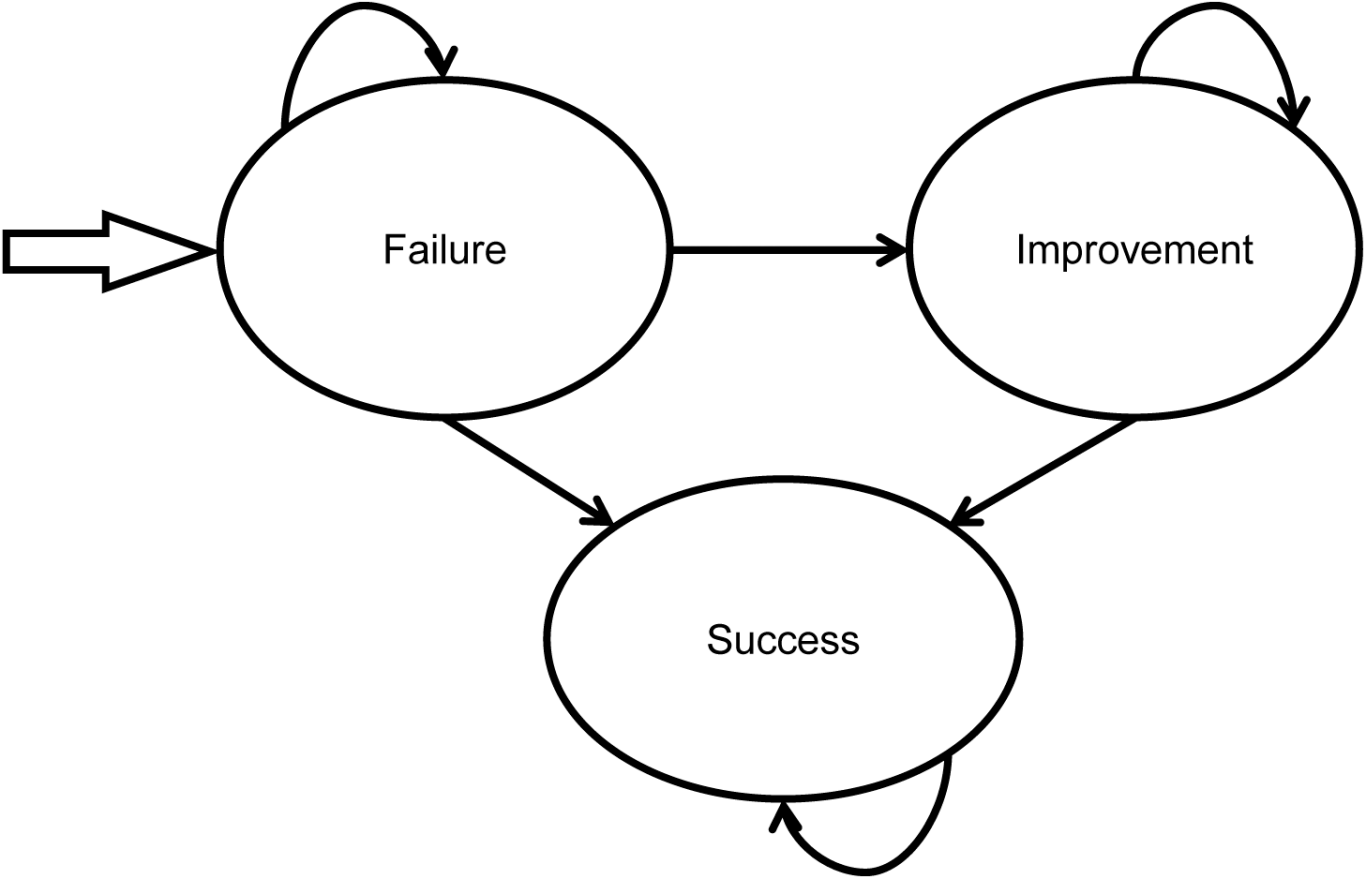
Markov model structure.

Patients who enter the treatment for cure pathway flow into a Markov process where they will move to one of three possible health states (Fig 2): 1) incontinent; 2) improvement, where patients experience at least 50% fewer incontinence episodes; and 3) success, where patients experience 100% fewer incontinence episodes. All patients begin the first treatment cycle in the incontinent health state. Each cycle in the model lasts 3 months. In the later cycles, patients can move from the incontinent health state to the improvement or success health states. Patients who enter the improvement health state can either remain in the improvement state or move to the success state. Patients who enter the success state remain in the state for the duration of the analysis.

**Fig 3:**
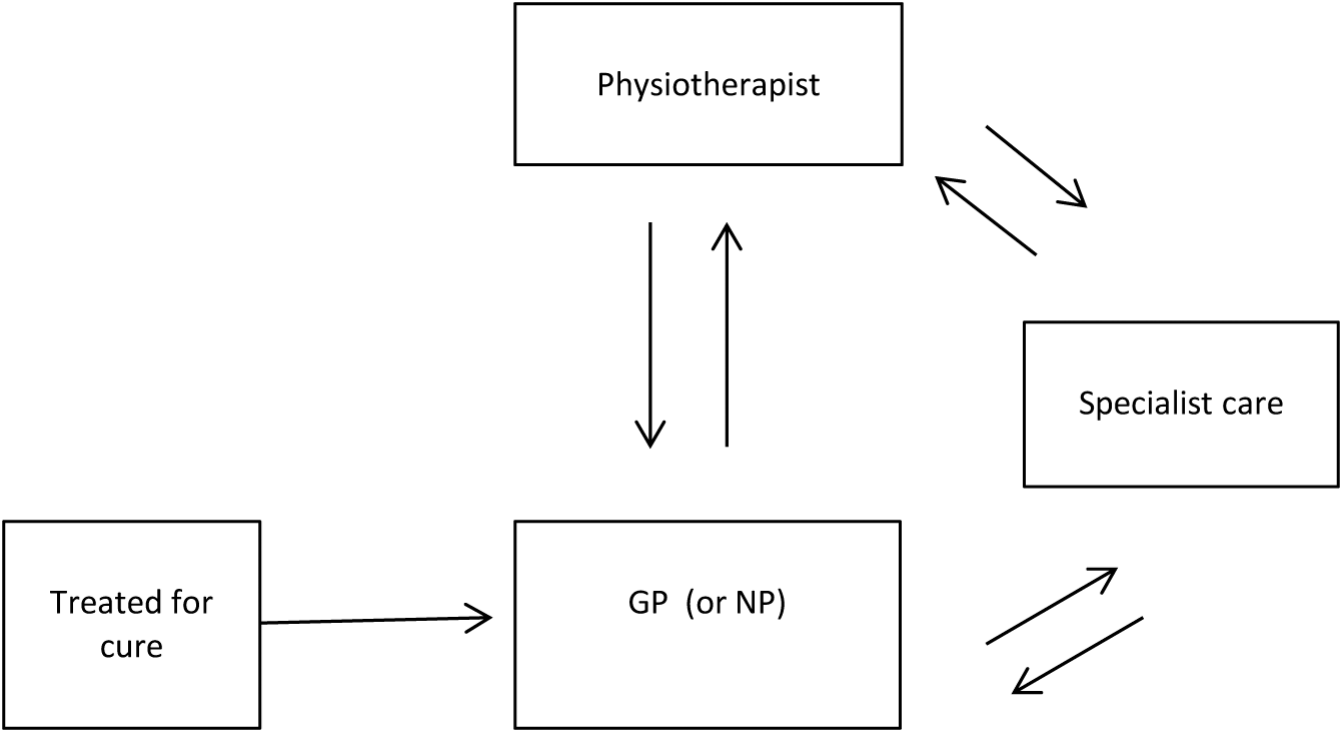
Care pathway for treatment for cure plus containment.

The probability of moving between health states in the treatment for cure pathway is dependent on the type of care a patient receives. Fig 3 describes the care pathway modelled on treatment for cure. In this pathway, all patients are initially treated in the primary care setting (i.e., first-line) by the GP. The GP then decides, based on the type and severity of UI, whether the patient should be referred immediately for pelvic physiotherapy or specialist care. Patients who are not referred immediately are then treated by the GP with one of the following therapy options: 1) medication; 2) lifestyle advice; 3) or treatment for urinary tract infection.

Patients who fail despite active treatment by the GP either remain incontinent and stop receiving active treatment or are referred to the pelvic physiotherapist or specialist for a second attempt at active treatment. Patients who are referred to the pelvic physiotherapist receive pelvic floor muscle training (PFMT) with or without biofeedback therapy. Patients who fail PFMT are subsequently referred either to a specialist or back to the GP. Patients who are referred to a specialist receive one of the following therapy options: 1) medication; 2) surgical procedure; 3) referral to the pelvic physiotherapist for eligible patients who have not received PFMT; 4) or other conservative therapy (for example, in the Netherlands occasionally pessaries are prescribed). All patients who fail specialist care are referred back to the GP.

Patients who enter the two care pathways for treatment by containment only and self-management are assumed to remain incontinent and hence remain in the incontinence health state. The only difference between these care pathways is that the costs of pad use for patients treated by containment are reimbursed by the health insurer in the Netherlands, thus representing costs borne by the health care system. The costs of pad use in the self-management pathway, however, are assumed to be borne by the patient and are included as out-of-pocket costs.

#### Model of current care versus new care

The model structure and patient flow are essentially the same for the current care and new care. However, in the new care strategy, the NS is assumed to be active in finding patients who are incontinent, thus increasing the detection rate. Also, instead of having an initial consultation with a GP or an immediate referral, patients now start with a consultation with the NS, who will perform an initial assessment, establish a diagnosis and start treatment on that basis. This may, or may not, include a prescription for containment and may be followed by a “containment only” strategy. In the case of treatment for cure, the NS will carry out three follow-up consultations that are used for training and life-style advice. After that, the NS may refer patients to the pelvic physiotherapist and the specialist. We assumed that from then on, the pathway does not differ from the current care strategy. The intervention has been described in more detail in S2 Appendix.

### Input parameters

#### Patient flow and effectiveness of current care

Table 2 provides details on the input parameters for transition probabilities used to model patient flow and the probability of treatment effectiveness in terms of improvement or success. Values for input parameters were taken from published literature wherever possible [32–36]; otherwise expert opinion was sought. Details from the expert interviews can be found in Table 3, and information on the search strategies applied in order to find relevant published studies can be found in S1 Appendix [37].

**Table 2:** Input parameters used to model patient flow and treatment effectiveness.

Input parameter	Current care	New care	Source
**Detection**			
% incident cases detected by GP	50,40%	64%	CC: Awareness study [45], NC:Assumption
Extra detection by NS		14%	Awareness study [45].
% prevalent cases never detected but incontinent	62,80%	48,79%	Derived with model
**Initial assessment**			
% incident cases assessed/diagnosed by GP	95%	100%	Assumption
% incident cases assessed/diagnosed by PPT	5%	0%	Assumption
**Treatment strategy**			
Treated for cure % incident cases	37%	39%	Difference
Treated for cure % prevalent cases	0,01%	6,67%	Derived with model
Containment only % incident cases	61%		Expert opinion
Containment only % prevalent cases	36,00%	44,54%	Derived with model
Self-management % incident cases	2%	0%	Assumption
Self-management % prevalent cases	1,20%	0,00%	Derived with model
*Active treatment in patients treated for cure*			
GP			
% of cases treated in cycle 1 by GP (incident population)	100%		Assumption
% of cases being treated in cycle 1 by GP (prevalent population)	33%		Assumption
Treatment decision by GP:			
Immediate referral	51%		Expert opinion and data.
To pelvic physiotherapist	57%		Expert opinion and data.
To specialist	43%		Expert opinion and data.
Immediate treatment attempt by GP	49%		Difference
% receive training from GP	0%		Expert opinion and data.
% training from GP with improvement	0%		Expert opinion and data.
% training from GP with success	0%		Expert opinion and data.
% receive medication from GP	79%		Expert opinion
% meds from GP with improvement	63%		Drutz et al. [32]
% meds from GP with success	16%		Imamura et al. [4]
% users medication that receive 2nd cycle medication*	76%		Sexton et al. [33]
% continue medications with improvement	80%		Imamura et al. [4]
% continue medications with success	20%		Drutz et al. [32]; Imamura et al. [4]
% receive lifestyle advice from GP	17%		Expert opinion
% lifestyle advice from GP with improvement	10%		Assumption
% lifestyle advice from GP with success	0%		Assumption
% receive treatment for urinary infection from GP	4%		Expert opinion
% treatment for infection from GP with improvement	40%		Assumption
% treatment for infection from GP with success	10%		Assumption
GP and NS			
% of cases treated in cycle 1 by GP/NS (incident population)		100%	Assumption
% of cases being treated in cycle 1 by GP /NS (prevalent population)		33%	Assumption
Percentage of cases initially treated by NS for 3 consults (i.e., first cycle)		96%	Difference
Cases treated initially by NS with improvement		21%	Subak et al. [18]
Cases treated initially by NS with success		31%	Subak et al. [18]
% of cases that initially receive treatment for urinary infection		4%	Expert opinion
Cases treated for infection by NS with improvement		80%	Assumption
Cases treated for infection by NS with success		20%	Assumption
% NP failures in cycle 1 continuing with NS care in cycle 2		40%	Assumption
% receiving medication from NS		100%	Expert opinion
Cases receiving meds from NS with improvement**		63%	Drutz et al. [32]
Cases receiving med from NS with success		16%	Imamura et al. [4]
Percentage users of medication under advice of NS that receive 2nd cycle of medication***		76%	Sexton et al. [33]
Cases that continue with meds in cycle 3 with improvement		80%	Drutz et al. [32]; Imamura et al. [4]
Cases that continue with meds in cycle 3 with success		20%	Drutz et al. [32]; Imamura et al. [4]
% of failures of NS care that are referred		60%	Assumption
To pelvic physiotherapist		33%	Assumption
To specialist		67%	Assumption
PT			
% cases treated in cycle 1 by PPT (incident population)	0%		Assumption
% cases being treated in cycle 1 by PPT (prevalent population)	33%		Assumption
Treatment decision by PPT:			
% receive PFMT training only	95%		Expert opinion
Cases receiving PFMT with improvement	62%	37%	Expert opinion.
Cases receiving PFMT with success	0%		Expert opinion
% receive PFMT plus biofeedback	5%		Expert opinion
Cases receiving PFMT plus biofeedback with improvement	45%		Burns et al. [34]
Cases receiving PFMT plus biofeedback with success	23%		Burns et al. [34]
% training patients with a success/improvement that continue	100%		Assumption
Cases of training patients in the second cycle with improvement	44%	46%	McFall et al. [35]
Cases of training patients in the second cycle with success	56%	54%	McFall et al. [35]
% of failures of PPT treatment that are referred	100%		Assumption
To GP	33%		Expert opinion
To specialist	67%		Expert opinion
Specialist			
% cases treated in cycle 1 by specialist (incident population)	0%		Assumption
% cases being treated in cycle 1 by specialist (prevalent population)	33%		Assumption
% cases referred immediately from specialist to PPT for training	40%		Expert opinion.
% cases treated immediately by specialist	60%		Difference
Treatment decision by specialist:			
% receive surgery	17%		Expert opinion
Cases receiving surgery with improvement	8%		Expert opinion.
Cases receiving surgery with success	77%		Labrie et al. [37]
% receive conservative therapy	2%		Expert opinion.
Cases receiving conservative therapy with improvement	34%		Richter et al. [36]
Cases receiving conservative therapy with success	0%		Assumption
% receive medication from specialist	81%		Expert opinion.
Cases receiving meds from specialist with improvement	63%		Drutz et al. [32]
Cases receiving meds from specialist with success**	16%		National Collaborating Centre for Women’s and Children’s Health & NICE [14]
% users of medication that receive 2nd cycle of medication***	76%		Sexton et al. [33]
Cases continuing meds with improvement	80%		Drutz et al. [32]; Imamura et al. [4]
Cases continuing meds with success	20%		Drutz et al. [32]; Imamura et al. [4]
% failures of specialist treatment that are referred	100%		Assumption
To GP	100%		Assumption
To pelvic physiotherapist	0%		Difference

GP: general practitioner, NS: continence nurse specialist, PPT: pelvic physiotherapist, PFMT: pelvic floor muscle training.

*Costs were valued based on the average daily dose of the following drugs used to treated urge UI: tolterodine, solifenacin, darifenacin, fesoterodine.

**Costs were valued based on average % of patients 'absolutely dry' after use of tolterodine IR, tolterodine ER, solifenacin, darifenacin, or fesoterodine.

***Costs were valued based on the average discontinuation rate from the studies that reported results at 9–12 months for the medications: tolterodine, solifenacin, darifenacin, fesoterodine.

**Table 3:** Probability inputs for current care based on expert opinion.

Parameters	Number of experts	Range	Value used in model
**Treatment strategy**			
Percentage of incident cases treated for containment only	3 GP and 1 database	33%-90%	61%
Cases treated for containment only with improvement	1 GP	0%	0%
Cases treated for containment only with success	1 GP	0%	0%
**Active treatment in patients treated for cure**			
GP			
Treatment decision by GP:			
Immediate referral	3 GP and 1 database	20%-100%	51%
To pelvic physiotherapist	3 GP and 1 database	47%-100%	57%
To specialist	3 GP and 1 database	0%-53%	43%
Percentage that receive training from GP	3 GP and 1 database	0%-1%	0%
Percentage that receive medication from GP	3 GP and 1 database	0%-100%	79%
Percentage that receive lifestyle advice from GP	3 GP and 1 database	0%-59%	17%
Percentage that receive treatment for urinary infection from GP			4%
PFPT			
Treatment decision by PFPT:			
Percentage that receive PFMT training only	1 PFPT	95%	95%
Cases receiving PFMT with improvement	2 PFPT	50%-80%	62%
Cases receiving PFMT with success	2 PFPT	0%	0%
Percentage that receive PFMT plus biofeedback	1 PFPT	5%	5%
Percentage of failures of PT treatment that are referred	Assumption		100%
Percentage of not improved patients referred back to GP	1 PFPT	33%	33%
Percentage of not improved patients referred back to specialist	1 PFPT	67%	67%
Specialist			
Percentage cases referred immediately from specialist to PPT for training	2 Gynaecologists and 2 Urologists	16%-80%	40%
Percentage cases treated immediately by specialist	2 Gynaecologists and 2 Urologists	20%-84%	60%
Treatment decision by specialist:			
Percentage receive surgery	2 Gynaecologists and 1 Urologist	17%	17%
Cases receiving surgery with improvement	1 Urologist*	8%	8%
Cases receiving surgery with success	1 Urologist + study Labrie et al [37]	78% + 76%	77%
Percentage receive conservative therapy	2 Gynaecologists and 1 Urologist	0%-4%	2%
Percentage receive medication from specialist	2 Gynaecologists and 1 Urologist	75%-100%	81%
			

GP: general practitioner; PPT: pelvic physiotherapist; PFMT: pelvic floor muscle training.

* Expert opinion was based on the average improvement rate of the ProAct and the TVT procedure

Some transition probabilities differ between incident and prevalent patients. Incident patients enter the care pathway at the start, whereas prevalent patients are already located somewhere in the pathway. In order to estimate the distribution of prevalent patients over the care pathway, we ran the model first with 100 hypothetical patients and checked where they were after 1 year (i.e., not detected, successfully treated for cure, treated by containment or self-care). Sixteen of these 100 patients had successfully been treated by that time and were no longer in the care pathway. The distribution of the remaining (now prevalent) patients is found in Table 3.

#### Effectiveness of nurse specialist-led care

In our search for the effectiveness of nurse-led incontinence care, we found 20 publications of studies, three of which were performed in a population that differed from our population (UI in younger patients, postnatal UI and men with UI after prostatectomy). In 9 of the 15 remaining studies an intervention comparable to the optimum continence service specification was compared with usual care (by GPs) [18, 38–44].

For the effectiveness of care provided by the NS we used the only study that reported the percentage of patients who became continent and the percentage of patients who improved by 50% [18]. No information was found in the literature regarding the increase of the detection rate. Therefore we used information from an international incontinence awareness study [45]. From that study, we assumed that the percentage of patients who stated that they had not visited their GP for incontinence problems but intended to do so in the future provided a reasonable estimate of the potential increase in detection with a NS. This increase in detected patients increases the pool of patients that may potentially be treated leading to success or improvement. As mentioned previously, one of the tasks of the NS would be to improve case coordination (i.e. liaise with formal home care agencies, with other health practitioners, and with informal caregivers) that may lead to improved effectiveness of new care, but probably also leads to increased costs. This task has, however, not been quantified in the analysis due to lack of evidence regarding its impact on health effects and costs.

#### Incontinence-related adverse events and use of formal home care and informal care

The costs and health impact of disease-related adverse events were included in the analysis alongside the use of medical and nonmedical home care support (Table 4). Disease-related adverse events included fractures, skin breakdown and urinary tract infections (UTI). The incidence of UTI was based on the difference between the yearly incidence of 1 or more episodes of UTI in patients with overactive bladder (OAB) compared with patients with no OAB (45.7%-15.4% = 30.3%), corrected for cycle length (8%) [15].

**Table 4:** Incidence of disease-related adverse events and use of medical and nonmedical support.

Input parameter	Estimate	Source
Incidence of disease-related adverse events		
Urinary tract infections (per 3 months)	8%	Hu and Wagner [15]
Fractures (per 3 months)	0.02%	de Rekeneire [46]; Hunter [47]; Meerding et al. [48]
Skin breakdown (per 3 months)	8%	Brown et al. [49]
Medical and nonmedical support needs		
Percentage users of informal care	43%	Langa et al.[50]
Percentage users of formal care	47%	Sorbye et al. [51]
% reduction use of formal or informal care in improved cases	10%	Assumption
% reduction use of formal or informal care in success cases	25%	Assumption

The incidence of fractures was derived from studies by de Rekeneire [46] and Hunter [47]. From these we estimated the percentage of falls attributable to incontinence per cycle (0.41%) and the percentage of falls resulting in a fracture (5.7%) [48], resulting in a percentage fractures per cycle of 0.023% (see S1 Appendix).

The incidence of skin breakdown was based on the findings of a 5% random sampling of the 1996–1997 California Medicaid Program (Medi-Cal) claims data in the U.S. which showed that 8% of the OAB population received treatment for skin infections [49].

To incorporate the costs and benefits of the new care in terms of informal care (i.e. care given by spouses, children, friends etc.) and formal home care (personal care, nursing care and home help by paid care giver), the percentage of patients requiring care were taken from two studies: a study by Langa et al. [50] in the U.S. which reported that 43% of elderly patients with UI require informal care, and a study by Sorbye et al. [51] at 11 sites in Europe which reported that 47% of UI patients require formal home care. Finally, to capture the cost-savings that result from the reduction in informal care and home care use for patients who move from the incontinence health state, we assumed that home patients experiencing improvement and success used 10% and 25% less care than other patients, respectively.

#### Costs

Costs were calculated from a societal and health care perspective. To calculate the total costs per comparator from the societal perspective all types of costs were included, i.e. medical treatment, consultations, containment products (both paid by insurer and out-of-pocket), incontinence-related adverse events such as fractures, skin breakdown and urinary tract infection, formal home care, informal care and travel costs (Table 5). Productivity costs (indirect non-medical costs) were not included since the target patient population was of retirement age. For the health care payer perspective, the informal care costs, travel costs and out-of-pocket costs for containment products were excluded. All costs were reported in terms of 2013 euros with historical prices corrected for inflation where necessary.

**Table 5:** Cost and utility parameters included in the model.

Input parameter	Base case value	Source
**Quality of Life**		
Utility in success health state	0.8595	Slieker-ten Hove et al. [58]
Utility in improvement health state	0.84205	Assumption
Utility in failure health state	0.8246	Slieker-ten Hove et al. [58]
**Direct medical costs (2013 euro)**	Cost per cycle	
Consultation GP	€ 30.48	Hakkaart-van Roijen et al. [52]
Consultation NS	€ 41.39	Salary per month (FWG 60, step 5) [53]
Cost medication use under care of GP or NS	€ 114.96	Health Care Institute [57]
Cost lifestyle advice under care of GP	€ 30.48	Hakkaart-van Roijen et al. [52]
Cost training under care of GP	€ 30.48	Hakkaart-van Roijen et al. [52]
Cost treatment of infections under care of GP	€ 2.51	National Health Care Institute. [57]
Consultation PPT	€ 39.19	Hakkaart-van Roijen et al. [52]
Cost PFMT training with PPT	€ 205.73	Expert opinion.
Cost training plus biofeedback/electro stimulation with PPT	€ 216.23	Expert opinion.
Consultation specialist	€ 130.62	Dutch Healthcare Authority (NZa). [56, 56]
Cost surgery under care of specialist	€ 317.97	Dutch Healthcare Authority (NZa). [56, 56]
Cost medication under care of specialist	€ 317.16	Dutch Healthcare Authority (NZa). [56, 56]
Cost conservative therapy under care of specialist	€ 137.74	Dutch Healthcare Authority (NZa). [56, 56]
Cost containment pads success cases	€-	Reimbursement price, Achmea [55]
Cost containment pads improved cases	€ 71.22	Reimbursement price, Achmea [55]
Cost containment pads failure cases	€ 97.70	Reimbursement price, Achmea [55]
Cost of treating UTI	€ 2.51	Hakkaart-van Roijen et al. [52]
Cost of surgery for fractures	€ 2,944.36	Meerding et al. [48]
Cost of skin breakdown	€ 6.49	Market price of Sudocrem
Cost of formal care, success cases	€ 2,894.53	Eggink et al. [54]; Reimbursement price, Achmea [55]
Cost of formal care, improved cases	€ 3,473.44	Eggink et al. [54]; Reimbursement price, Achmea [55]
Cost of formal care, failure cases	€ 3,859.38	Eggink et al. [54]; Reimbursement price, Achmea [55]
**Direct nonmedical costs (2013 euro)**		
Travel costs GP/NP	€ 3.51	Hakkaart-van Roijen et al. [52]
Travel costs PPT	€ 13.94	Hakkaart-van Roijen et al. [52]
Travel costs Specialist	€ 4.79	Hakkaart-van Roijen et al. [52]
Cost informal care success cases	€ 1,596.75	Hakkaart-van Roijen et al. [52]
Cost informal care improved cases	€ 1,916.09	Hakkaart-van Roijen et al. [52]
Cost informal care failure cases	€ 2,128.99	Hakkaart-van Roijen et al. [52]
**Indirect costs (2013 euro)**		
Out-of-pocket costs of containment pads	€ 97.70	Reimbursement price, Achmea [55]
Implementation cost of new care per year	€ 426,496.00	See S2 Appendix

GP: general practitioner, PPT: pelvic physiotherapist, NS: continence nurse specialist, UTI: urinary tract infections.

Costs are generally estimated by combining resource use with a cost per unit of care. The Dutch costing manual was used to value the costs of travel, hours of formal home care, hours of informal care and consultations [52], with the exception of NS consultations, which were based on a pro-rated hourly wage [53]. To calculate the hourly wage for NSs, an annual salary of €44,736, which was based on an average monthly salary of €3,728 (Functional scale 60, step 5), was divided by 1,540, which are the average number of workable hours per year assuming a 36-hour week.

The average weekly use of informal care was taken from a US study on informal caregiving time in a group of patients with pad use [50], which was corrected for the proportion of males and females in the target patient population. Formal home care was estimated based on the average number of hours of personal home care for the elderly in the Netherlands [54, 55]. Where possible, the costs of medical treatment and incontinence-related events were taken from publicly available tariffs made available from the Dutch Healthcare Authority (NZA) [56] or the National Health Care Institute (former CVZ) [57], and otherwise taken from published literature. The costs of containment pads for patients in the incontinent and improvement health states were valued based on the reimbursement price and average daily use which was adapted according to a scale for severity of UI symptoms and the corresponding daily pad use [55]. Patients in the success health state were assumed to have no or negligible pad use. Out-of-pocket costs for containment products for incontinent patients who were never detected and patients who manage their incontinence with self-care were included and were based on the costs applied to incontinent patients.

For new care we also included implementation costs, which were based on the cost to educate and train 500 NSs calculated on the basis of 4000 GP locations in the Netherlands with 1 NS visiting 8 locations per week. We assumed that the total training costs can be depreciated over a period of 5 years. For the base case analysis, we included 23.8% of the total training costs, as the target population of the current analysis reflects approximately 23.8% of the total UI and FI population in the Netherlands (see S2 Appendix) and, once trained NS are working within a GP practice, they will also take on the care for other UI and FI patients. However, in a scenario analysis we explored how the results change when all implementation costs are assigned to the current target population.

#### Health benefits

Health benefits were expressed in quality-adjusted life years (QALYs), where a quality of life weight is given to each of the 3 health states defined. To calculate QALYs, utility values were applied to the time patients spent in the various health states of success, improvement and incontinence (Table 5). The utility values applied in the model were taken from a cross-sectional study of quality of life using a EQ-5D questionnaire in a general female population aged 45–85 in the Netherlands [58]. The utility of patients with improvement was based on the average of the utility of patients with success (continent) and the utility of patients with UI.

### Budget impact analysis

For the budget impact analysis, the population considered consists for the first year of all prevalent and incident patients. In the second year, new incident cases are added, whilst patients already in the pathway may exit the model either because they die or they move to a nursing home. The same applies to the third year: new cases flow into the pathway, other patients are removed from the pathway. Mortality was estimated using Dutch life tables [29]. From these we found the probability of dying in the subsequent 1, 2 or 3 years for patients in all the relevant age bands. Unfortunately we did not have data specific to patients with at least 4 chronic diseases, so the mortality rates used for the budget impact analysis are likely to be a slight underestimate.

The percentage of patients moving to a nursing home per year was estimated at 4% (see S2 Appendix for the calculation).

### Assessment of uncertainty

The influence of parameter uncertainty on the results was assessed by one-way sensitivity analysis (OWSA) and probabilistic sensitivity analysis (PSA). In the OWSA, each parameter was varied within a range of plus or minus 40% of the mean value. A PSA was performed to assess the level of confidence surrounding the decision in which the values of all parameters are simultaneously allowed to vary. For the PSA, cost parameters were allowed to vary within a range of plus or minus 20% of the mean value. A beta distribution with a standard error equal to 20% of the mean was applied in the PSA for all parameters limited to the interval of 0 and 1, such as transition probabilities and utilities. For all other parameters, a uniform distribution was assumed. In a separate PSA we also used a percentage of 40% instead of the more commonly used 20% in order to account for the low level of evidence used in this model (e.g. the parameter estimates that were based on the expert opinion).

### Scenario analysis

A scenario analysis was conducted to assess the impact of assuming that the full 100% rather than 23.8% of the implementation costs are included in the costs of new care.

In addition, we explored the impact of the effectiveness of the NS in two scenarios: 1) new care has impact only on the detection rate and not on the effectiveness of the care; 2) new care has impact only on the effectiveness of care but not on detection.

## Results

### Base case

The analyses showed that for the incident patient group, 9% of patients will be successfully treated in the usual care pathway, versus 14% of patients in the new care pathway (see Table 6). In addition, the percentage of patients that is improved increases by 3%. For the prevalent patients, the extra detection of patients by the NS leads to 4% of patients now being successfully treated and 3% of patients showing an improvement.

**Table 6:** Outcomes, percentages successfully treated and improved patients.

	Incident group			Prevalent group		
	Usual care	New care	Incremental	Usual care	New care	Incremental
% success	9%	14%	5%	0%	4%	4%
% improved	8%	11%	3%	0%	3%	3%
% not improved	84%	76%	-8%	100%	94%	-6%

Looking at costs and health benefits, we see that the new care strategy is cost-saving compared with usual care, with an average savings of €402 per patient from a societal perspective (Table 7). When the perspective is limited to the health care payer perspective, an average cost saving of €97 per patient is achieved over the three year time frame. At the same time, on average a small gain in quality of life (0.005) is achieved.

**Table 7:** Costs and QALYs per patient per 3 years.

	Usual care	New care	Difference
GP (+NS)	€ 20	€ 47	€ 27
Pelvic physiotherapist	€ 14	€ 13	€ -1
Specialist	€ 25	€ 26	€ 0
Containment (insured)	€ 436	€ 563	€ 127
UI-related adverse events	€ 16	€ 16	€ -1
Home care	€ 21,576	€ 21,323	€ -253
Implementation costs	€ -	€ 5	€ 5
Total health care costs	**€ 22,088**	**€ 21,991**	**€ -97**
Out-of-pocket costs*	€ 700	€ 523	€ -177
Informal care costs	€ 10,889	€ 10,761	€ -128
Total societal costs	**€ 33,677**	**€ 33,276**	**€ -402**
Total QALYs	**2.4777**	**2.4829**	**0.005**

*Note that over 99.9% of out-of-pocket costs are for containment products. The remaining costs are travel costs.

Overall, the increased percentages of successfully treated and improved patients reduce the costs of formal home care, informal care and containment products. However, some of the costs for containment are now shifted from out-of-pocket costs for the patient to costs for the health insurer.

### Budget impact

In addition to the per patient analysis, we also looked at the budget impact of the optimum continence service specification on a national level. Over a 3 year period, the total savings would amount to € 29 million from a payer perspective and € 117 million from a societal perspective (see Table 8).

**Table 8:** Budget impact over a period of 3 years.

	New care	Usual care	Incremental
Societal perspective	€ 9,505 M	€ 9.622 M	- € 117 M
Health care payer perspective	€ 6.282 M	€ 6.311 M	- € 29 M

M = Million

### Sensitivity analysis

**Fig 4:**
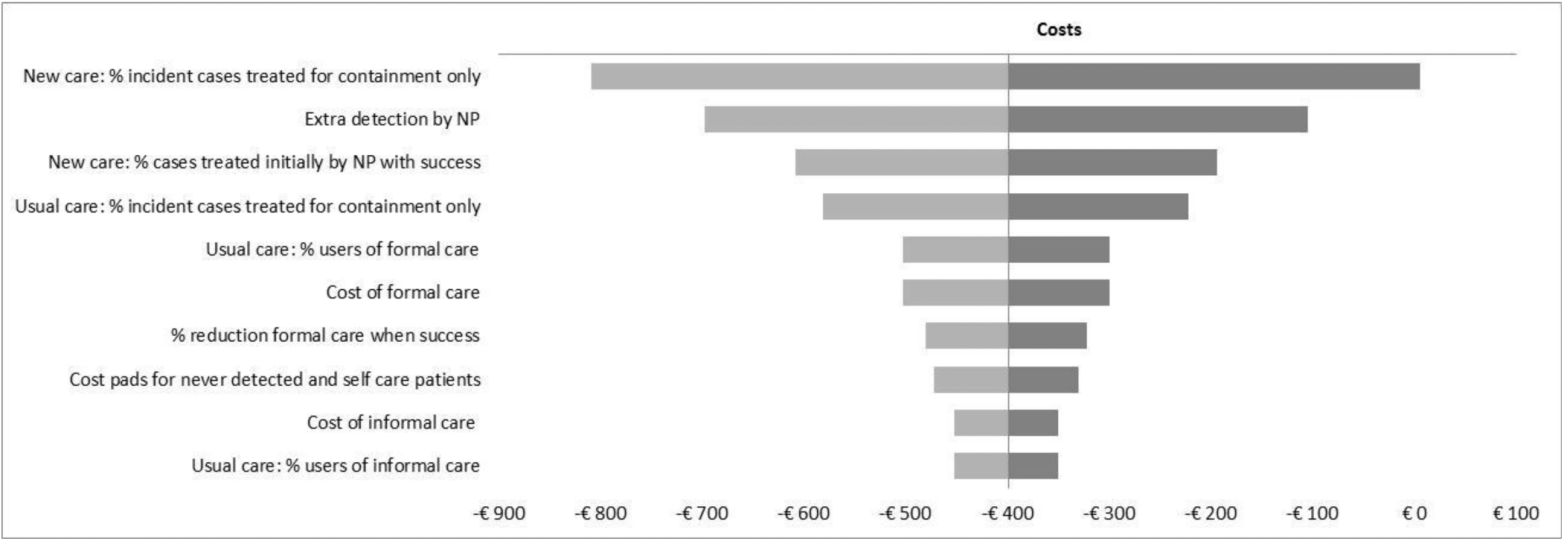
Diagram depicting the impact of influential parameters on the cost-savings.

Results from the OWSA are presented in Fig 4, showing the impact of influential parameters on the estimate for incremental costs. While the range of incremental costs consistently fall below zero, i.e., cost-saving, the magnitude of cost-savings is influenced by a number of parameters. The most influential parameters include estimates associated with the effectiveness of new care. Cost-savings are increased as the percentage of patients receiving treatment for cure by the NS increases, the detection rate of UI patients attributable to the NS increases, and the success rate of NS care increases. Other influential factors include the costs and frequency of use of both formal and informal care in current care and the out-of-pocket costs of containment products for patients who manage their incontinence with self-care. The influence of direct costs outside the health care sector underscores the need to consider the societal costs when performing economic evaluations of UI care.

The results of the PSA (with a SE of 20% of the mean) show a 95% range of incremental costs from a saving of €911 to additional costs of €41, with a 96% probability of cost savings. For incremental QALYs, the 95% range varies from -0.0005 to 0.013, and a 5% probability of fewer QALYs. Also, the PSA results show that, with 95% certainty, the new care intervention dominates current care i.e. it is more effective and cost-saving. For the PSA with increased uncertainty (with a SE of 40% of the mean) we found an 80% probability of cost saving and a 22% probability of fewer QALYs. The overall probability that the new care intervention would dominate the current care was now 75%.

### Scenario analyses

The impact of the assumption that 100% rather than 23.8% of the implementation costs are included in the costs of new care was shown to be of little influence on the cost-savings. The results for cost-savings were slightly lower compared with the base case at €387 and €83 from the societal and healthcare perspective, respectively.

When we assume that new care has impact only on the detection rate and not on the effectiveness of the care, we find that the QALYs gained reduce to 0.004 whilst from a societal perspective costs are still saved (€-203). From a health care perspective we now observe a cost increase of € 58. In that latter situation, the ICER is €14,500 per QALY gained. When we look at the reverse scenario in which the NS has an impact on the success rate but is not able to detect more patients the QALYs gained reduce to 0.001, with a cost saving of €109 and €65 from a societal and health care perspective, respectively.

## Discussion

Our study has shown that with the new care strategy, with a nurse specialist located in primary care, a small gain in quality of life is achieved. This increase in quality of life is achieved at reduced cost compared with usual care, with an average saving of €402 per patient over a 3 year period from a societal perspective. When the perspective is limited to the health care payer perspective, an average cost saving of €97 per patient is achieved over the three year time frame. In interpreting these findings it is important to realise that many patients are undetected, even in the new care situation (36%), or receive treatment for containment only. In both of these groups it is assumed that no cure or improvement of the UI will be achieved.

The results of our study are based on the effectiveness of a NS in the RCT by Subak et al. [18]. Previous studies have shown conflicting evidence for the benefits of a nurse-led intervention for UI. A meta-analysis of 11 randomised trials comparing nurses with GPs showed no benefit in terms of health outcomes, although little data on the cost impact were available in these trials [59]. More recent studies of nurse-led continence care were conducted alongside randomised controlled trials. Two were conducted in the UK [60, 61] and the third in the Netherlands [62]. The earlier study from the UK presents findings that conflict with those used in the present study. Moore et al. [60] studied standardised conservative therapy regimens provided by nurse continence advisors versus urogynaecologists in 110 consecutive women with mild or moderate urinary leakage. The authors found that the reduction in urine leakage and improvement in quality of life observed in patients treated by nurse continence advisors and urogynaecologists were similar at 12 weeks and 2 years but that lower costs arose from treatment provided by nurse advisors. The conflicting results for health benefits could be explained by the difference in the comparator used. In the present study the nurse led intervention was compared with the care as usual by the GP who, according to interviews with experts, in general does not provide PFMT and bladder training. In the study of Moore et al. [60], the urogynaecologists did provide these. Also, due to the high drop-out rate of their study (24%), the authors encouraged caution in the interpretation of their findings. A more recent study in the UK by Williams et al. [61] evaluated the impact of a new service led by a continence nurse practitioner compared with existing primary/secondary care provision for men and women aged 40 or above with urinary incontinence and storage symptoms. The authors found that the continence nurse practitioner-led intervention improved health outcomes through reduced symptoms at 3 and 6 months; impact was reduced; and satisfaction with the new service was high. At 6 years of extended follow-up, the health benefits of the nurse-led intervention group remained significant compared with the control group [42]. Williams et al. [61] found the costs to increase with nurse-led care. However, they did not include the costs of formal home care and informal care, which we have shown to be an influential factor in realising cost-savings. Findings from a study performed in the Netherlands by Albers-Heitner et al. [62] demonstrated that although a nurse-led intervention increased QALYs compared with care-as-usual, it was more expensive due to the additional implementation costs. These findings were, however, limited to 12-month outcomes. Extending the timeframe of the analysis beyond one year, as was done in the present study, could produce different results if the implementation costs were assumed to be one-off costs for investing in the new care approach. More importantly, just as in the study of Williams et al (2011), Albers-Heitner [62] did not include the costs of formal home care and informal care in which a cost-reduction is expected. The costs of incontinence-related adverse events were also not taken into account. Comparison between the results presented here and that of previous studies should be viewed carefully since the methodology, type of nurse-led intervention and target patient populations are not entirely comparable.

The potential effects of a nurse-led intervention for UI related to improvement in tailoring prescribed containment products to the patient’s and caregiver’s’ need and a potential improvement in case coordination have not been taken into account in the current analysis. Although one of the authors mentioned above reported some information on the proportion of patients receiving special information about adequate use of containment products by a NS, this study did not provide enough information to estimate its effect on the costs and quality of life per health state [63]. An improved tailoring of containment products could either increase or decrease the costs for containment products but could be expected to increase a patient’s quality of life and reduce the work load for informal care givers. In addition, the presence of a NS who can coordinate care between the various health care professionals may lead to either an increase or decrease of resource use. In the event of an increase, this would be likely to be associated with a gain in quality of life for some patients.

In our study, health effects were expressed in quality-adjusted life years. This means that utility weights were assigned to the health states: incontinent, improved and success (i.e. continent). Our values were taken from a survey among people aged between 40 and 80 years. Thus, these utilities may not be fully applicable to our population of older persons with at least 4 co-existing morbidities. However, for the calculation of QALYs gained with new care the decrement of people with incontinence versus continence is more important than the absolute value. In various studies with interventions in incontinence utilities have been measured and, regardless of the intervention (medication, surgery, physiotherapy), a similar decrement to the one applied here was observed [64–66]. It should be realised that there may be gains for patients beyond those measured in generic quality-of-life instruments such as the EuroQol EQ-5D [67]. For example, incontinence may hinder patients in carrying out activities such as volunteer work or informal care giving. If patients can be socially active when they become continent again or better manage their incontinence, society as well as the individual will benefit.

The sensitivity analysis demonstrated a few key parameters contributing to the uncertainty surrounding the results. While it was shown that the results for cost-savings were generally robust, the magnitude of possible cost-savings is greatly influenced by the assumptions made regarding the effectiveness of new care and the need for medical and non-medical home care support. The impact of introducing a nurse specialist into the care of UI patients in the Netherlands is unknown both in terms of its effectiveness and the use of formal home care and informal care. Given the potential for cost-savings and the possibility of achieving savings of up to €911 per patient, further research into the impact of a NS both on patient outcomes and resource use in the treatment of UI in the Netherlands would be worthwhile.

An important limitation of our current model of the care pathway is that we assumed that patients who had achieved a complete or partial success would stay in that health state for the duration of the model. Given the age of the population considered in our study, it seems likely that over time some patients may become incontinent again. However, a recent review of the long term effectiveness of pelvic floor muscle training showed that at least a substantial proportion of patients remained continent or in their improved state. Given the relatively short time horizon of our model, the impact of a small group of patients becoming incontinent again is likely to be small [68].

This relatively short time horizon of our model might be another limitation of our study. Although none of the patients still received treatment at the end of our 3-year time horizon, and thus the large majority of costs and health effects are captured in our model, it will probably not capture all costs and health effects. Patients who did not become continent after the first three years, will probably keep using containment products and some patients who became continent might become incontinent again and vice versa (Kondo et al). Since information about relapse rates and spontaneously becoming continent was not available for our population, we considered a longer time horizon highly speculative.

The current evaluation only considered one subset of all people with incontinence. Once continence nurse-specialists are introduced in primary care, they can of course also take on the care for other incontinent patients, and it might be interesting to explore the economic impact of this. This is especially true for the group with faecal or double incontinence. However, we found that the feasibility of adapting the model used in this study to the care of FI was limited by the complete lack of any studies regarding a nurse-led intervention for FI. In addition, the care pathway is more complex (using a multi-component approach) for these patients and it is less clear what constitutes a (partial) success in treatment [1].

It is important to realise that the current results are specific for the Netherlands, where GPs function as gatekeepers to specialist care and thus play a key role in the treatment of incontinence. A number of options were considered for the choice of the healthcare professional taking the lead role. In the Netherlands there are varying levels of continence training for nurses. The introduction of continence nurse specialists, rather than advanced practice nurses or other relevant clinical providers may be a more expensive model but it was felt that this level of nurse would provide the greatest clinical benefit and best fitted the Netherlands health service.

In countries where nurse specialists play a more important role in patient care or staff with an equivalent specialist continence training or other relevant background are unavailable, other treatment pathways will need to be defined and the activities of the healthcare professionals specified. The number of patients treated by GP, specialist or pelvic physiotherapist may differ, resulting in different costs and potentially also different outcomes. This also means that the best person to take on the coordinating role may vary between countries, which may in turn affect both the additional costs and the achieved effects of implementing the changes recommended by the optimum continence service specification.

It should be realised that, in the Netherlands, the role of continence nurse specialists is still quite new, with few nurses having completed this training. Thus, it will take a few years before these continence NS can start to deliver continence care on a large scale.

In conclusion, implementing the optimum continence service specification in the Netherlands by having a continence nurse specialist in the GP practice is likely to reduce the level of incontinence, improve quality of life, and reduce costs—from a payer’s perspective as well as from the patient’s and carer’s perspective. The total budget savings may even increase as the population ages. At the same time, the current study has shown that various areas of the incontinence care process lack data, and thus it would be important to introduce the continence nurse specialists in a study setting.

## Supporting Information

S1 Appendix Literature search strategy (DOCX)

S2 Appendix Calculation of input parameters (DOCX)
