# Supplementary material for: Cost-Effectiveness of Including a Nurse Specialist in the Treatment of Urinary Incontinence in Primary Care in the Netherlands
Source: PLoS One. 2015 Oct 1;10(10):e0138225. doi: 10.1371/journal.pone.0138225 (PMC4591337; doi:10.1371/journal.pone.0138225)
Supplement: S1 Appendix — (DOCX) [file pone.0138225.s001.docx]

## S1 Appendix. Literature search strategy

To ensure all available information was reviewed when choosing the input parameters for the model, systematic literature searches were conducted in PubMed. The searches focused on the costs and success rates of continence care and the utilities of patients with incontinence. Reference lists were also screened to identify additional references.

One of the following incontinence search terms were used in every search and had to appear in the title: incontinence, incontinent, continence, continent and overactive bladder. In the search for effectiveness of the optimum service specification [1] the following search terms were used as well: incontinence nurse, continence nurse, specialised nurse, specialised nurse, POH, behavioral nurse, nurse, continence advisor, consultant, management, nurse led, treatment, conservative, behavioural, model, care combined with the following search terms primary care, community, dwelling, community-dwelling. These studies were also screened for information about costs of incontinence or continence care.

Information about the effectiveness of pelvic physiotherapy was searched using the search terms: pelvic floor muscle training, pelvic floor muscle exercise therapy, pelvic floor electrical stimulation, pelvic floor muscle exercise, pelvic floor exercise, physiotherapy, physical therapy, behavioral training, behavioral therapy, behavioral management, biofeedback, muscle contraction, exercise therapy, conservative treatment, conservative measures, rehabilitation, health education, and bladder training combined with search terms for older patients (i.e. elderly, elder, older, old, aged, menopausal, postmenopausal).

Finally, studies reporting quality of life were identified using the following search terms: SF-36, SF-6D, EQ-5D, HUI, quality of wellbeing, disability adjusted life years, quality adjusted life years, (health related) quality of life, time tradeoff, standard gamble, visual analog scale, health state, disutility and utility.

Considering the changes in healthcare, studies published before 2000 may not be representative for the current health care system, therefore those articles were excluded. Articles had to be written in English language. The titles and abstracts of the studies obtained by the searches were screened for appropriateness for this study. If the article seemed relevant the entire article was read and where possible relevant data was extracted.

References

1. Wagg AS, Newman DK, Leichsenring K, van Houten P. (2014) Developing an internationally-applicable service specification for continence care: Systematic review, evidence synthesis and expert consensus. PLoS One 9: e104129.
