# Supplementary material for: Cost-Effectiveness of Including a Nurse Specialist in the Treatment of Urinary Incontinence in Primary Care in the Netherlands
Source: PLoS One. 2015 Oct 1;10(10):e0138225. doi: 10.1371/journal.pone.0138225 (PMC4591337; doi:10.1371/journal.pone.0138225)
Supplement: S2 Appendix — (DOCX) [file pone.0138225.s002.docx]

S2 Appendix. Calculation of input parameters

Incidence of urinary tract infections

According to the study of Wagner et al. [1] patients with overactive bladder (OAB) have on average 0.457 UTI’s per year compared to 0.154 UTI’s in patients without OAB. This means that on average 0.303 UTI’s per year are a consequence of OAB, which translates into a probability of 8% to get UTI per cycle.

Incidence of fractures (per cycle)

The incidence of fractures was derived in two steps. First the percentage of falls due to incontinence was estimated en then the percentage of falls resulting in a fracture was determined.

For the first step the cross-sectional study by de Rekeneire [2] was used among people from 70 to 79 years living in the community. This paper reported an odds-ratio of 1.5 for falling in incontinent versus continent people. This odds-ratio was adjusted for various factors. In the study, the probability of falling for continent people was 18% and thus, a relative risk of falling for incontinent versus continent people was derived of RR= 1-18% + 18% * 1.5 = 1.09. So, corrected for other factors, the extra percentage of falls in incontinent patients would be 1.09*18% - 18% = 1.6%, over a period of 1 year.

The probability that a fall results in a fracture was derived from Hunter [3]. In this study women with lower urinary tract symptoms were followed for falls. In total 35 falls occurred, of which 2 resulted in a fracture. Combining this with the probability of falling, we find per year 0.093% patients with a fracture attributable to incontinence, which is 0.023% per 3-month cycle. Finally, these fractures were split between wrist (90%) and hip (10%) fractures according to Meerding et al. [4].

Incidence of skin infections (per cycle)

Urine or faecal leakage and its subsequent prolonged contact with skin could lead to skin irritation and infections. Brown et al. [5] (page S575) found that 8% of the OAB population received treatment for skin infections. Based on this finding it is assumed that 8% of the patients in our study get a skin infection.

Medication

Costs of medication

The costs of the drugs were obtained from the website of the National Health Care Institute (www.medicijnkosten.nl). The dosage was based on the average of the lowest and highest dosage stated in the NHG standard ‘Incontinence for urine’. Patients were assumed to be equally distributed over the following drugs: tolterodine (oral), tolterodine (oral prolonged release), solifenacin, darifenacin and fesoterodine (€0.845 per day). The total costs of medication per cycle consist of the costs of two consultations with a specialist (based on NZA tariffs) and the costs of medication for three months.

Percentage of patients with improvement or success from medication provided by GP/NS/Specialist

In the NICE guideline the mean continence status probability is derived from 20,000 random simulations in a network meta-analysis. In our model the average of these probabilities for the included drugs was calculated to obtain the success rate of medication (Table 6.19, 16%) [6].

The percentage of patients with improvement from medication was not available for every drug included in this study. Therefore the improvement rate in the model was based on the reduction in frequency of incontinence caused by tolterodine (63%) [7], since according to a recent study of Ju et al. [8] tolterodine was the most commonly reported medication for women with overactive bladder in the United States (33.80%).

Percentage users of medication GP/NS/Specialist that receive medication in a subsequent cycle

Sexton et al. [9] found that rates of treatment discontinuation are consistently high. Most studies report discontinuation rates at 12 weeks. However, expert opinion revealed that many patients do not adhere to their medication after 12 months. Therefore we calculated the average discontinuation rate (24%) from the studies that reported results at 9-12 months for the medications included in our study. The percentage of patients that continue using medication is therefore 76%.

Percentage of patients with improvement or success from medication provided by GP/NS/Specialist in 2^nd^ cycle

For the effectiveness of the subsequent cycle of medication use, we assumed that all patients who continued using medication after the first cycle experience some level of improvement. For the distribution over the improved and success state we used the ratio between improvement: 63% [7] and success: 16% [6] (80% vs. 20%).

Treatment by NS during 3 consultations

In the new care situation the nurse specialist (NS) is responsible for active identification of patients who are incontinent, but not detected by the GP. All identified patients with urinary incontinence will be invited for a one hour consult where the type of incontinence will be assessed. During the assessment a clinical history and a physical examination will be undertaken. This includes mid-stream specimen of urine; estimation of post-void residual volume; blood pressure; body mass index; and in women, a vaginal examination. The patients are instructed to use a voiding diary. During the second visit (30 minutes) the voiding diary will be reviewed and treatment will be determined and started. Possible treatments provided by the continence nurse include advice on diet and fluids; bladder training; pelvic floor awareness and healthy eating; treatment for candida and urinary tract infection where indicated. During the third visit (30 minutes) advice and therapy will be reinforced. During the last visit the effectiveness of the intervention will be assessed. Individuals whose symptoms persisted after this primary intervention are seen by the general practitioner to discuss the desirability of referral to a specialist or pelvic physiotherapist.

Implementation costs

To become a NS, nurses have to complete the Master of Advanced Nurse Practice (two years). The total costs of this master are €17.920; including tuition fees, costs of the counseling practice educator, tax deductible, and book money and the cost of attending an international conference (https://www.han.nl/opleidingen/masters/advanced-nursing-practice).

We assumed one NS could treat patients in 8-10 primary care practices. This means we would need 500 NSs in the Netherlands. The total costs of education are therefore €8.96M. This amount was spread out over 5 years as a one-time investment in capital (similar to that for equipment). Since not only the patient group in our model is expected to benefit from the presence of a NS in the GP practice but all patients with UI and FI, only 23.8% of the costs was attributed to our current target population.

This all results in a total cost of 426,496 euros per year for the UI patients in our study population. This is multiplied by 3 for a time horizon of 3 years.

The 23.8% estimate was calculated as follows. In the NIVEL database, a total of 867,140 patients are included. In that population, the prevalence of UI is 16.1 per 1000 and of FI 1.7 per 1000, resulting in a total of 15695 patients with UI or FI. In the same database, a total of 3736 patients above 65 years old with 4 multiple morbidities and UI are included, resulting in 3736/15695=23.8% of all UI and FI patients**.**

Extra detection by NS

It is assumed the NS will take a pro-active role in the detection of patients with UI or FI. The increased detection rate is based on the general awareness study in which patients from Germany, Sweden and the UK reported whether they contacted a healthcare provider for diagnosis [10]. The detection rate is derived from the proportion of patients (14%) in the UK that did not contact a healthcare provider yet, but intend to do so. It is expected these patients could be reached by the NS.

Percentage of cases with improvement and success

The effectiveness of the NS is based on the study of Subak et al. [11]. Although the nurse-led behavioural therapy was not completely comparable with our intervention, we used information about the effectiveness of this intervention, since this study was the only study available that reported the proportion of patients who were cured (improvement of 100%) and improved at least 50%. After this behavioural therapy 31% of the women were cured and 21% (52%-21%) of the women were at least 50% improved [11].

Treatment by pelvic physiotherapist

The pelvic physiotherapist can give pelvic floor muscle training (PFMT) with and without biofeedback. Expert opinion revealed 5% of the patients receive PFMT with biofeedback; consequently 95% receive PFMT without biofeedback. Expert opinion estimated that treatment exists of 9 to 12 therapy sessions on average. It is assumed patients receive 10.5 therapy sessions over 6 months and therefore 5.25 therapy sessions in one cycle.

Costs of pelvic physiotherapy

The consultation costs of the pelvic physiotherapist are obtained from the Dutch handbook for costing studies [12]. Expert opinion revealed PFMT with biofeedback has an additional cost of €21 per patient. These costs are spread over the 6 months of treatment and are therefore €10.50 per patient per cycle.

Percentage of patients receiving pelvic floor muscle training without biofeedback with success or improvement after one cycle

The percentage of initial improvement after pelvic floor muscle training (PFMT) without biofeedback was based on expert opinion.

In the new care situation, the treatment of the NS partially covers the PFMT training by the pelvic physiotherapist. Since it is likely the NS will only refer ‘complicated’ cases to the pelvic physiotherapists - the relatively ‘easy’ patients can be successfully treated by the NS – the success and improvement rates were adjusted.

Percentage of patients receiving pelvic floor muscle training plus biofeedback with success or improvement after one cycle

The improvement and success rates of PFMT combined with biofeedback were based on the study of Burns et al. [13]. The results of this study showed that in 23% of the patients the urine loss improved with 100% and in 45% of the patients the urine loss improved with at least 50% (Table 2, page M171).

Percentage of patients receiving pelvic physiotherapy with success or improvement after two cycles

The study of McFall et al. [14] studied the outcomes of a small group educational intervention for urinary incontinence in older women (65 years or older). The results showed that 35% of the patients experienced a 100% reduction, and 27% experienced at least 50% reduction in the number of incontinence episodes.

For the effectiveness of the subsequent cycle of treatment by a pelvic physiotherapist in the current situation, we assumed that all patients who continued treatment after the first cycle experience some level of improvement. For the distribution over the improved and success state we used the ratio between improvement (27%) and success (35%): 44% vs. 56%.

Treatment by specialist

Costs of surgery

Information of the Dutch Healthcare Authority (NZA) was used to calculate the average costs of surgery for incontinence.

Percentage of patients receiving surgery with success or improvement

According to the opinion of experts’ opinions only patients with pure stress urinary incontinence receive surgery. The tension-free vaginal tape (TVT) was the most reported surgery performed in these patients. The results of the study of Labrie et al. [15] show that 76.5% of patients were objectively cured (defined as a negative provocative cough stress test) by the TVT surgery. The expert opinions did not provide information about the probability of men receiving surgery; therefore we assumed this was the same as the probability for women. The proportion of patients with at least 50% improvement was based on expert opinion.

Costs of conservative treatment

The costs of these conservative treatments are based on the NZA tariff of outpatient treatment for incontinence.

Percentage of patients receiving conservative treatment with success or improvement after one cycle

The improvement rate is based on the study of Richter et al. [16] that showed that 34.2% had at least 75% reduction in weekly urinary incontinence episodes. We assumed that the cure rate was 0%.

Percentage of patients going to nursing home (budget impact analysis)

We based our estimation on the assumption that each nursing home bed stays occupied for approximately 2 years. Assuming a total of 80,000 beds (currently there are more beds in the Netherlands, but new policies aim to reduce this to 80,000), the number of admissions per year would be 40,000. From these, approximately 50% of patients is incontinent, i.e. 20,000. The overall population of people over 65 is 2,600,000, and with 19% prevalence of UI [17] the total number of elderly with incontinence comes to 494,000. So we see 20,000 out of 494,000 patients, i.e. 4%, being admitted to a nursing home each year. Note that we assume that this estimate also applies for patients with at least 4 multiple morbidities, though in reality these patients may be more likely to be admitted.

Impact of treatment on formal and informal home care

The population of community-dwelling patients older than 65 years with four or more chronic diseases was defined using a published list of chronic diseases based on ICPC-2 codes (but recoded to ICPC-1) [18]. This list comprises 146 classification codes of the ICPC-2 list. We know from previous studies that the most common chronic diseases among elderly in the general population are: hypertension, osteoarthritis, ischemic heart disease, obesity, atherosclerosis and diabetes [19]. These diseases do not require (formal) home care in a substantial part of the elderly, even when multiple diseases are present, but can also require daily formal home care. An average of 25% of homecare to be due to incontinence may therefore not be an unrealistic assumption.

References

1. Wagner TH, Hu TW, Bentkover J, LeBlanc K, Stewart W, Corey R, et al. Health-related consequences of overactive bladder. Am J Manag Care. 2002;8: S598-607.

2. de Rekeneire N, Visser M, Peila R, Nevitt MC, Cauley JA, Tylavsky FA, et al. Is a fall just a fall: Correlates of falling in healthy older persons. the health, aging and body composition study. J Am Geriatr Soc. 2003;51: 841-846.

3. Hunter KF, Voaklander D, Hsu ZY, Moore KN Lower urinary tract symptoms and falls risk among older women receiving home support: A prospective cohort study. BMC Geriatr. 2013;13: 46-2318-13-46.

4. Meerding WJ, Mulder S, van Beeck EF Incidence and costs of injuries in the netherlands. Eur J Public Health. 2006;16: 272-278.

5. Brown JS, McGhan WF, Chokroverty S Comorbidities associated with overactive bladder. Am J Manag Care. 2000;6: S574-9.

6. National Collaborating Centre for Women’s and Children’s Health & National Institute for Health and Care Excellence (NICE)Urinary incontinence in women: The management of urinary incontinence in women. Royal College of Obstetricians and Gynaecologists. 2013. Available: [http://www.nice.org.uk/guidance/CG171](http://www.nice.org.uk/guidance/CG171).

7. Drutz HP, Appell RA, Gleason D, Klimberg I, Radomski S Clinical efficacy and safety of tolterodine compared to oxybutynin and placebo in patients with overactive bladder. Int Urogynecol J Pelvic Floor Dysfunct. 1999;10: 283-289.

8. Ju CC, Swan LK, Merriman A, Choon TE, Viegas O Urinary incontinence among the elderly people of singapore. Age Ageing. 1991;20: 262-266.

9. Sexton CC, Notte SM, Maroulis C, Dmochowski RR, Cardozo L, Subramanian D, et al. Persistence and adherence in the treatment of overactive bladder syndrome with anticholinergic therapy: A systematic review of the literature. Int J Clin Pract. 2011;65: 567-585.

10. Bacher, J. Awareness about incontinence among the general public. Paper presented at the 5ft Global Forum on Incontinence: Better Care, Better Health -Towards a Framework for Better Continence Solutions. Madrid. Available: [http://www.gfiforum.com/Documents/Madrid-2014/Conference%20Summary%20Report%205th%20GFI.pdf](http://www.gfiforum.com/Documents/Madrid-2014/Conference%20Summary%20Report%205th%20GFI.pdf).

11. Subak LL, Quesenberry CP, Posner SF, Cattolica E, Soghikian K The effect of behavioral therapy on urinary incontinence: A randomized controlled trial. Obstet Gynecol. 2002;100: 72-78.

12. Hakkaart-van Roijen L, Tan SS, Bouwmans CAM.(2011) Handleiding voor kostenonderzoek, methoden en standaard kostprijzen voor economische evaluaties in de gezondheidszorg. geactualiseerde versie 2010. Diemen: Department FIA CVZ.127p.

13. Burns PA, Pranikoff K, Nochajski TH, Hadley EC, Levy KJ, Ory MG A comparison of effectiveness of biofeedback and pelvic muscle exercise treatment of stress incontinence in older community-dwelling women. J Gerontol. 1993;48: M167-74.

14. McFall SL, Yerkes AM, Cowan LD Outcomes of a small group educational intervention for urinary incontinence: Health-related quality of life. J Aging Health. 2000;12: 301-317.

15. Labrie J, Berghmans BL, Fischer K, Milani AL, van der Wijk I, Smalbraak DJ, et al. Surgery versus physiotherapy for stress urinary incontinence. N Engl J Med. 2013;369: 1124-1133.

16. Richter HE, Burgio KL, Brubaker L, Nygaard IE, Ye W, Weidner A, et al. Continence pessary compared with behavioral therapy or combined therapy for stress incontinence: A randomized controlled trial. Obstet Gynecol. 2010;115: 609-617.

17. Teunissen TA, van den Bosch WJ, van den Hoogen HJ, Lagro-Janssen AL Prevalence of urinary, fecal and double incontinence in the elderly living at home. Int Urogynecol J Pelvic Floor Dysfunct. 2004;15: 10-3; discussion 13.

18. O'Halloran J, Miller GC, Britt H Defining chronic conditions for primary care with ICPC-2. Fam Pract. 2004;21: 381-386.

19. Schram MT, Frijters D, van de Lisdonk EH, Ploemacher J, de Craen AJ, de Waal MW, et al. Setting and registry characteristics affect the prevalence and nature of multimorbidity in the elderly. J Clin Epidemiol. 2008;61: 1104-1112.
